# BLOOD PRESSURE MEASURES ARE NOT CORRELATED WITH NIH TOOLBOX COGNITIVE BATTERY PERFORMANCE IN BLACK OLDER ADULTS

**DOI:** 10.1093/geroni/igad104.2620

**Published:** 2023-12-21

**Authors:** Allison Moll, Colt Halter, Hannah Adams, Loraine DiCerbo, Voyko Kavcic, Ana Daugherty, John Woodard, Bruno Giordani

**Affiliations:** Wayne State University, Detroit, Michigan, United States; Wayne State University, Detroit, Michigan, United States; Wayne State University, Detroit, Michigan, United States; Wayne State University, Detroit, Michigan, United States; Wayne State University, Detroit, Michigan, United States; Wayne State University, Detroit, Michigan, United States; Wayne State University, Detroit, Michigan, United States; University of Michigan, Ann Arbor, Michigan, United States

## Abstract

Elevated blood pressure has been inconsistently associated with cognitive performance in older adults. Black adults experience higher rates of elevated blood pressure and cognitive impairment than White adults. This study investigated whether blood pressure measurements were associated with cognitive performance on the NIH Toolbox Cognition Battery (NIHTB-CB) in older Black adults. 77 older Black adults 65+ years of age (M=72.6, SD=4.9) with subjective cognitive decline were administered the NIHTB-CB, a battery of computer-based cognitive tests. Systolic blood pressure (SBP) and diastolic blood pressure (DBP) were measured before and after cognitive testing and a cognitive task-based EEG/ERP. Average SBP, DBP, and pulse pressure (PP) were calculated. Difference scores from pre- to post-EEG/ERP served as a reactivity index following cognitive task engagement. A ratio of the difference scores to the baseline measures controlled for resting blood pressure. Bayesian correlations examined the relations between blood pressure measurements and NIHTB-CB performance. Data analysis used fully corrected T scores from the NIHTB-CB subtests. Average SBP, SBP difference scores, and baseline SBP were not associated with NIHTB-CB performance on any measurement (all BF10 < 3.0). Similarly, DBP and PP were unrelated to NIHTB-CB performance. Systolic and diastolic blood pressure were not associated with any NIHTB-CB measure. Additionally, blood pressure reactivity was not related to cognitive performance. Future research may investigate the relationship between SBP reactivity and cognitive performance across multiple measurement periods. Antihypertensive medication and duration of hypertension among hypertensive individuals may also be an important consideration in future studies.

